# Temporal profiling of myocardial inflammation and recovery in a murine model of cardiac arrest and adrenaline exposure

**DOI:** 10.1016/j.resplu.2025.101137

**Published:** 2025-10-17

**Authors:** Soumya Panigrahi, Siyi Jiang, Angela Enriquez, Sanjana Tummala, Donald Rempinski, Kenneth E. Remy, Cody A. Rutledge

**Affiliations:** a Blood, Heart, Lung, and Immunology Research Center, Department of Medicine, Case Western Reserve University School of Medicine, Cleveland, OH, USA; b Division of Cardiology, Vascular Medicine Institute, Department of Medicine, University of Pittsburgh, Pittsburgh, PA, USA; c Department of Medicine, Louis Stokes Cleveland VA Medical Center, Case Western Reserve University School of Medicine, Cleveland, OH, USA; d Department of Medicine, Pittsburgh VA Medical Center, Pittsburgh, PA, USA

**Keywords:** Cardiac arrest, Inflammation, Heart, Mouse

## Abstract

**Background:**

Myocardial dysfunction after cardiac arrest (CA) is a major contributor to poor outcomes, yet the underlying inflammatory mechanisms within cardiac tissue remain incompletely defined. We sought to delineate the temporal evolution of myocardial inflammation following CA, distinguishing the effects of ischemia–reperfusion injury from those of adrenaline exposure alone.

**Methods and Results:**

Using a murine model of CA and resuscitation, we profiled transcriptomic, immunologic, and functional cardiac changes in mice exposed to either CA followed by resuscitation with adrenaline (Arrest group), adrenaline alone (Adr group), or anesthesia alone (Naïve group). Animals were assessed at 0.5-, 1-, 3-, and 7-days via echocardiography, RNA-sequencing, flow cytometry, immunohistochemistry, and multiplex cytokine analysis. CA elicited a robust, early myocardial inflammatory response characterized by neutrophil infiltration, systemic cytokine surges (IL-6, TNF-α, IL-1β), and downregulation of mitochondrial and metabolic pathways. This immune activation peaked at 0.5 days and resolved by day 7, coinciding with transient myocardial dysfunction and recovery of ejection fraction. In contrast, adrenaline alone induced a delayed, attenuated response peaking at 1 day. Transcriptomic and immunophenotypic signatures distinguished ischemia-driven injury from catecholaminergic effects.

**Conclusions:**

CA induces a distinct, self-limited myocardial inflammatory cascade that parallels functional cardiac recovery. These findings identify a narrow therapeutic window for immunomodulatory interventions and provide a mechanistic foundation for targeted therapies to mitigate cardiac injury after resuscitation.

## Introduction

Cardiac arrest (CA) remains a leading cause of sudden mortality worldwide. Despite improvements in emergency response and access to prompt cardiopulmonary resuscitation (CPR), CA continues to pose significant clinical challenges.^1^, ^2^, ^3^ Among survivors, CA induces widespread molecular and metabolic dysregulation that promotes systemic inflammation and immunological injury.^4^, ^5^, ^6^ Currently, no widely adopted therapies effectively improve long-term outcomes after CA.

CA initiates a complex cascade of systemic events that impact multiple organs, termed post-cardiac arrest syndrome (PCAS), and often affects myocardial function.^7^, ^8^ During CA, tissues experience severe oxygen and nutrient depletion, leading to ischemia, hypoxia, ATP depletion, and acidosis.^9^ Resuscitation reintroduces oxygen abruptly, generating reactive oxygen species (ROS) and oxidative stress, causing impairment of calcium handling and mitochondrial function, reduced ATP synthesis, and activation of inflammatory and cell death pathways.^9^, ^10^, ^11^, ^12^ Adrenaline, while essential for resuscitation, further contributes to myocardial disruption by promoting expression of inflammation-related genes and cytokine release.^13^, ^14^ Together, oxidative stress, calcium dysregulation, metabolic derangements, and inflammation can cause transient contractile dysfunction, commonly termed myocardial stunning.

Systemic inflammation is a hallmark of PCAS, and elevated inflammatory markers are associated with poor neurologic and cardiac outcomes.^15^, ^16^ Infiltrating immune cells, well characterized in myocardial infarction,^17^ likely play a central role in the post-CA myocardial response. Recent studies have utilized modern transcriptomic analyses to better understand inflammatory changes by studying circulating blood in cardiac arrest patients^18^ and by characterizing neuroinflammatory changes in a porcine model.^19^ Despite this recent focus of neuro-specific inflammatory changes and a broader interest in systemic inflammation in PCAS, the local immune and transcriptomic landscape *in the heart of* post-CA patients remains poorly defined. The timing, magnitude, and composition of cardiac immune cell infiltration, and how this differs from adrenaline-mediated effects, are not well characterized. Moreover, the temporal resolution of inflammation and its correlation with myocardial recovery are critical to identifying therapeutic windows.

In this study, we employed a murine model of CA and controlled adrenaline exposure to delineate the myocardial inflammatory trajectory post-resuscitation. We hypothesized that ischemia–reperfusion injury, rather than adrenergic stimulation alone, is the dominant driver of myocardial immune activation. Through comprehensive temporal profiling using echocardiography, transcriptomics, flow cytometry, histology, and cytokine analyses, we aimed to define the molecular and cellular basis of post-arrest cardiac inflammation and identify candidate targets for intervention.

## Materials and methods

### Cardiac arrest model

**Fig. 1:**
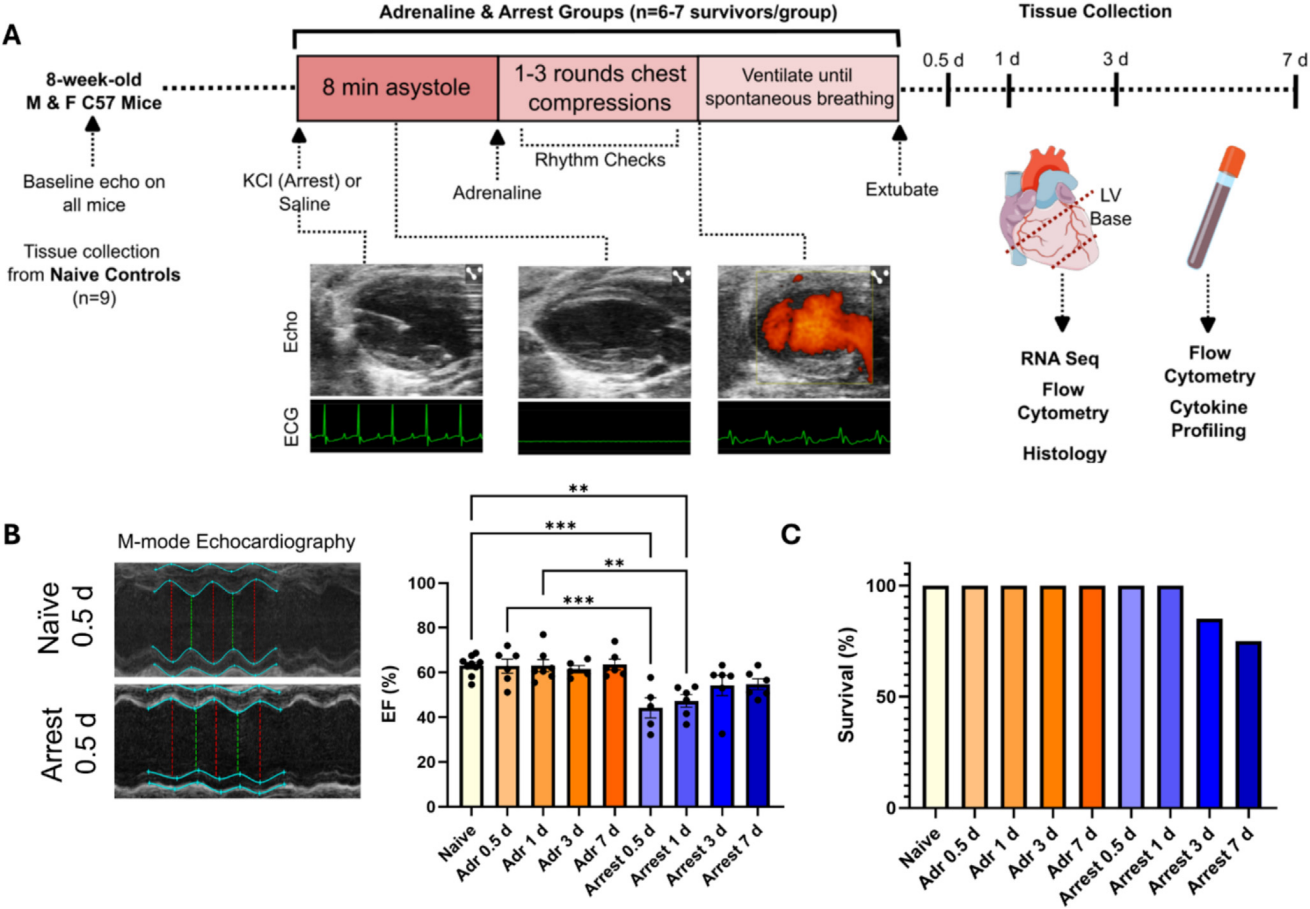
**Mouse model of cardiac arrest.** A. Study overview depicting potassium chloride (KCl) injection to cause asystole with representative ECG data during normal sinus rhythm, asystole, and resuscitation, as well as description of end-points for tissue collection. B. Representative M−mode echocardiography (left panel) in Epi and Arrest (0.5d after procedure). Ejection Fraction (EF) in Naïve, Adr, and Arrest groups at study end points (right). C. Percent of all animals surviving to study endpoint. *n* = 6–9/group. **=*p* < 0.005, ***=*p* < 0.0005 by ANOVA with Dunnett’s multiple comparison test.

Eight- to ten-week-old male and female C57BL/6J mice (Jackson Lab, Bar Harbor, ME) were assigned to Naïve, Adrenaline (Adr), or Arrest groups. All mice were anesthetized with 5 % isoflurane (Henry Schein, Melville, NY) prior to procedure. Adr and Arrest mice were intubated and mechanically ventilated. Mice then underwent ultrasound-guided, percutaneous LV injection of 40 µL of either saline (Adr group) or 0.5 M potassium chloride (Arrest group; Fig. 1)^20^ Naïve mice did not receive injection and were not intubated. Asystole was confirmed by ECG and doppler flow analysis of LV flow in the Arrest group. 8 min after saline injection (Adr) or asystole (Arrest group), 500 µL of adrenaline in warmed saline (15 μg/mL) was injected intraventricularly, and chest compressions promptly initiated in the Arrest group. Return of spontaneous circulation (ROSC) was defined by restoration of sinus rhythm on ECG with confirmatory doppler analysis of LV blood flow. Mice that did not achieve ROSC within 3 min were euthanized (total of 4 mice throughout the study). Surviving mice remained ventilated until spontaneous respiration resumed and were recovered under heat.

### Treatment duration, echocardiography, and tissue collection

Baseline systolic function was assessed in all groups by transthoracic echocardiography (Vevo 3100, Visual Sonics, Toronto, Canada), maintaining heart rate at 400–500 bpm.^21^ Naïve mice were euthanized after imaging, followed by tissue collection. Adr and Arrest mice recovered for 0.5-, 1-, 3-, or 7-days, followed by repeat echocardiography, euthanasia, and tissue collection. 6–9 mice were included for each time point studied, with additional mice included in the 3-and 7-day groups to account for mortality (1 mouse died post-operatively in the 3-day Arrest group, 2 mice in the 7-day Arrest group). After recovery, blood was collected, hearts were flushed with cold saline, and the apex was discarded to avoid local needle injury. LV tissue was flash-frozen or enzymatically digested for flow cytometry. All procedures were approved by the Pittsburgh VA Medical Center IACUC (#1680736).

### Transcriptomics data and immunoprofiling

RNA was isolated from 50 mg LV tissue (RNeasy Mini Kit, Qiagen), and quality was confirmed via NanoDrop and TapeStation (≥1.8 260/280 ratio, RIN ≥ 6, *n* = 3–5 per group). Bulk RNA-seq was performed by Novogene (Illumina NovaSeq PE150). Reads were processed with FastQC and aligned to the mouse genome using HISAT2 v2.0.5. One poor-quality Adr sample (3-day) was excluded. Differentially expressed genes (adj. *p* ≤ 0.05, log_2_FC > 0) were identified using DESeq2. Reactome pathway analysis (adj. *p* < 0.05) and immune gene cross-referencing were used for immunoprofiling (Supplemental Table 2).^22^ Z-scores for 16 immune populations were compared across groups.^23^ Heatmaps and PCA plots were generated using Heatmapper^24^ and ClustVis.^25^ RNA-seq data are available via GEO (GSE280586).

### Flow cytometry

Heart bases were minced and digested in DMEM with collagenase I, hyaluronidase, and DNase I (Sigma) at 37 °C for 1 h (*n* = 3–5 per group). Suspensions were filtered (40 µm), centrifuged (400 g, 5 min), subjected to ACK lysis, and resuspended in FACS buffer. Cells were stained with antibodies (Supplemental Table 1) for 30 min at 4 °C. Spleens were similarly processed without digestion. Whole blood underwent ACK lysis and staining. Samples were analyzed on a Cytek Aurora using FCS Express 7 (Denovo Software; Supplemental Figs. 4–6).

### Histology

Formalin-fixed, paraffin-embedded transverse heart sections (8 µm) were cut near the base (Pitt Histology Core, *n* = 4 per group). Sections were incubated with goat anti-MPO (1 µg/mL; R&D Systems), followed by HRP-conjugated anti-goat IgG and DAB staining. Slides were counterstained with hematoxylin. Images (40x, EVOS 5000) were analyzed by a blinded observer using ImageJ to quantify neutrophils per high-power field.

### Cytokine analysis

Serum cytokines (IFNγ, IL-2, IL-6, TNF-α, IL-1β, IL-17, GM-CSF, CCL3) were analyzed using custom ELLA cartridges (ProteinSimple, San Jose, CA) using 25 uL of plasma according to manufacturer recommendation and analyzed using Simple Plex Runner v3.7.2.0 (*n* = 3–5 per group).

### Statistical analysis

Data are presented as mean ± standard error unless otherwise noted. Significance was set at *p* ≤ 0.05. One-way ANOVA with Dunnett’s test was used for group comparisons, except for Fig. 4, which used nested ANOVA with Tukey’s test. Adr/Arrest time points were compared to Naïve and to each other. Analyses were done with GraphPad Prism 8 (San Diego, CA).

## Results

### Cardiac contractility is transiently reduced following CA

Naïve (anesthesia only), Adr (adrenaline administration), and Arrest mice (8 min of asystole followed by adrenaline mediated resuscitation; Fig. 1A) were assessed for cardiac contractility both at baseline and post-operatively (Adr and Arrest groups only) at 0.5-, 1-, 3-, and 7-days. Mice in the Arrest group exhibited a significant decrease in ejection fraction (EF) at 0.5 days [44.2 ± 4.5 %, *p* < 0.0005] and 1-day [47.3 ± 4.5 %, *p* < 0.005] post-resuscitation compared to Naïve mice [63.1 ± 1.5 %; Fig. 1B]. EF was also reduced in Arrest mice compared to time-matched Adr controls at 0.5 days [44.2 ± 4.5 % Arrest vs 62.9 ± 3.1 % Adr, *p* < 0.0005] and at 1-day [47.3 ± 4.5 % Arrest vs 63.1 ± 2.7 % Adr, *p* < 0.005; Fig. 1B]. By day 3, EF had returned to near baseline levels in the Arrest mice [54.2 ± 4.5 %], indicating recovery from myocardial stunning. No significant changes in left ventricular end-diastolic volume (LVEDV) or temperature were observed across groups (Table 1). Adr animals maintained normal systolic function throughout all time points. These findings validate the reproducibility of our CA model and confirm transient myocardial dysfunction specific to ischemia–reperfusion injury, rather than adrenergic stimulation alone. Survival to endpoint was 100 % in the Naïve and Adr groups, while minor attrition occurred in Arrest animals at later time points (1/7 at day 3, 2/8 at day 7, Fig. 1C). In the Arrest group, mean time to ROSC was 0.3 min, and time to extubation approximately 22 min (Table 1).

**Table 1:** **Physiologic and survivorship data across Naïve, Adrenaline (Adr) and Arrest groups.** Left Ventricular End Diastolic Volume (LV EDV) is the volume in the left ventricle at the end of relaxation. Return of spontaneous circulation (ROSC) indicates the amount of time elapsed between 8-min asystole time and the first observed ventricular complex by ECG and confirmation with Doppler flow analysis. Time to extubation is the total duration of time from procedure start to extubation. Data expressed ± standard deviation. Weights are reported from the day of procedure. Temperatures are reported 1 h after procedure. *Animal death post-op day 2 (1 female), **Animal death post-op days 1 and 2 (both males).

	**Number of Mice Studied (Total)**		**Weight (g)**		**LV EDV (µL)**		**Temp after procedure (°C)**		**Time to ROSC (min)**	**Time to extubation (min)**
**Naïve**	**9 (9)**		**22.6 ± 3.0**		**62.9 ± 6.0**		**n/a**		**n/a**	**n/a**
	**Adr**	**Arrest**	**Adr**	**Arrest**	**Adr**	**Arrest**	**Adr**	**Arrest**	**Arrest**	**Arrest**
**0.5 d**	6 (6)	6 (6)	24.5 ± 2.7	22.7 ± 2.3	70.7 ± 12.7	71.5 ± 9.6	36.7 ± 0.4	36.5 ± 0.6	0.22 ± 0.05	21.3 ± 1.8
**1 d**	7 (7)	7 (7)	23.5 ± 2.7	22.1 ± 2.7	59.3 ± 13.0	61.8 ± 5.4	36.5 ± 0.2	36.8 ± 0.5	0.50 ± 0.16	22.3 ± 2.5
**3 d**	6 (6)	6 (7)*	22.5 ± 2.6	21.9 ± 2.3	69.5 ± 20.5	57.8 ± 12.3	36.8 ± 0.2	36.5 ± 0.4	0.26 ± 0.05	22.4 ± 1.1
**7 d**	6 (6)	6 (8)**	23.6 ± 3.6	21.4 ± 2.2	54.1 ± 6.8	63.5 ± 18.5	36.8 ± 0.1	36.1 ± 0.2	0.23 ± 0.03	21.0 ± 1.6

### Transcriptomic profiling reveal early and transient pathway alterations following CA

Understanding the dynamic molecular responses to cardiac arrest is essential for identifying early regulatory mechanisms and potential therapeutic targets. Here, transcriptomic profiling uncovers rapid and transient alterations in key pathways, shedding light on the immediate genomic shifts that define the acute phase of cardiac injury and recovery. To identify global transcriptional changes in response to cardiac arrest and adrenaline exposure, we performed bulk RNA sequencing of myocardial tissue harvested at 0.5-, 1-, 3-, and 7-days post-intervention across all groups.

**Fig. 2:**
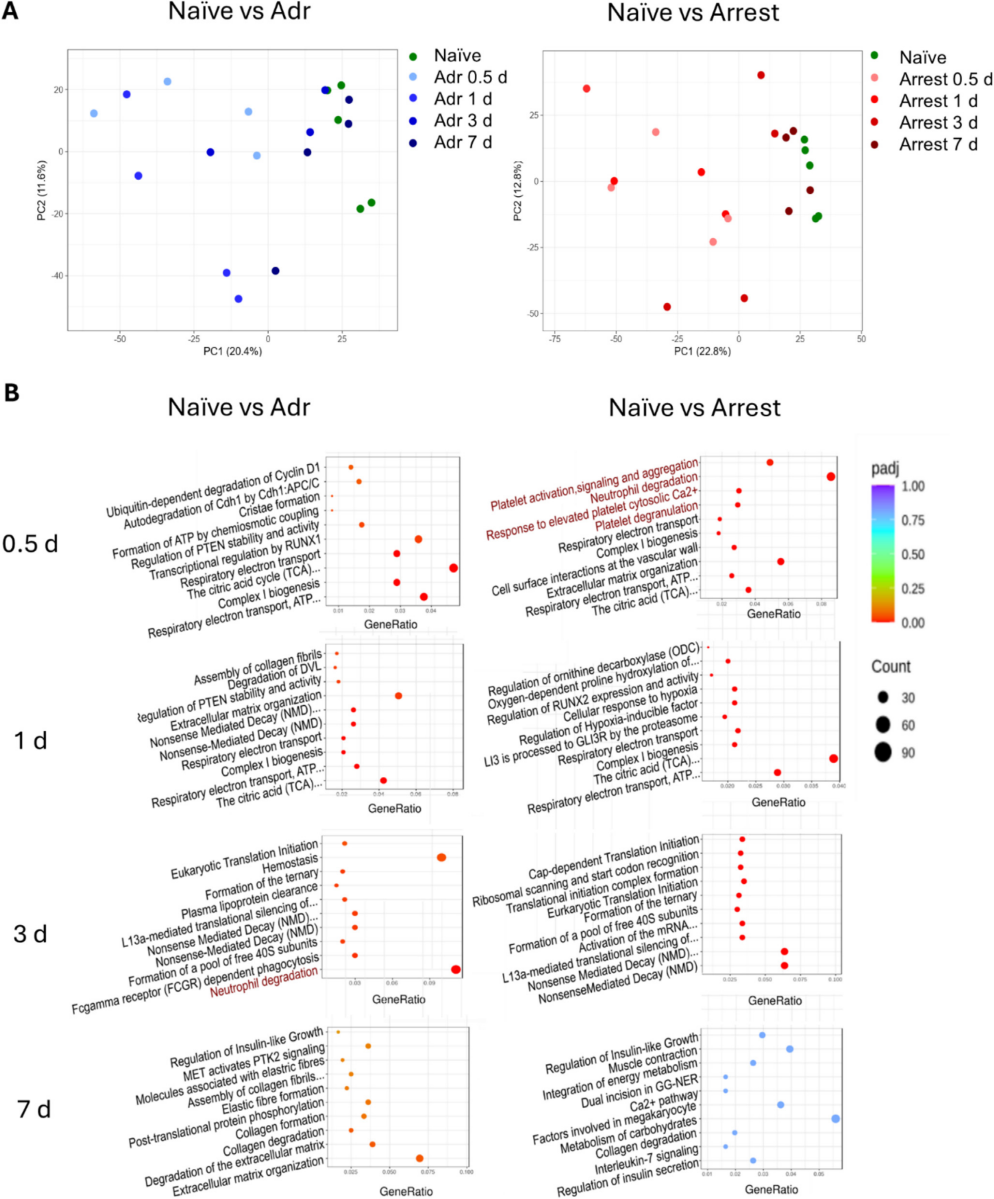
**Transcriptomic comparison and pathway Analysis of Epi and Arrest myocardial changes to Naïve controls.** A. Principal component analysis (PCA) plots of Naïve transcriptomes compared to Adr (left) or Arrest (right) at 0.5-, 1-, 3-, and 7-d, demonstrating resolution of changes over time. B. Top 10 Reactome pathway analyses comparing Naïve controls to Adr (left) and Arrest (right) hearts at study end-points. Inflammation associated pathways are highlighted in red. (For interpretation of the references to colour in this figure legend, the reader is referred to the web version of this article.)

Principal component analysis (PCA) revealed distinct clustering by experimental group and time point (Fig. 2A). Arrest animals demonstrated the most pronounced transcriptomic deviation at 0.5 days, which resolved progressively by day 7, paralleling recovery of cardiac function. In contrast, the Adr group exhibited a milder transcriptional response, with delayed peak changes at 1 day.

Reactome pathway analysis was utilized to identify broad categories of transcriptome changes (Fig. 2B). The top 10 Reactome pathways are presented for each time point by adjusted p-value, allowing comparison of Naïve controls to both Adr and Arrest animals. The most prominent pathways in both the Adr and Arrest groups predominantly involve mitochondrial and energetic pathways at 0.5- and 1-day (e.g., citric acid cycle, respiratory electron transport, complex I biogenesis). The Arrest mice, however, feature a number of inflammatory pathways (e.g. platelet activation, neutrophil degranulation) at 0.5 days, which are not present at later time points. Of note, the Adr groups do contain some inflammation associated pathways (e.g. neutrophil degranulation) at the 1-d end-point, though these pathways do not fall into the top ten changes (see Supplemental Table 3 for full list). By 7 days, there were minimal pathway changes in either group. No pathways were significantly different between Naïve and Arrest mice at 7 days, while only three pathways (related to extracellular matrix and collagen degradation) remained significant between Adr and Naïve mice at this time point, suggesting a near-complete resolution of transcriptomic changes. Pathway comparisons between Adr and Arrest groups directly revealed only a few significant alterations, most notably involving extracellular matrix organization at 0.5 and 1 day (Supplemental Fig. 2).

### Immunoprofiling reveals dynamic inflammatory responses following cardiac arrest

**Fig. 3:**
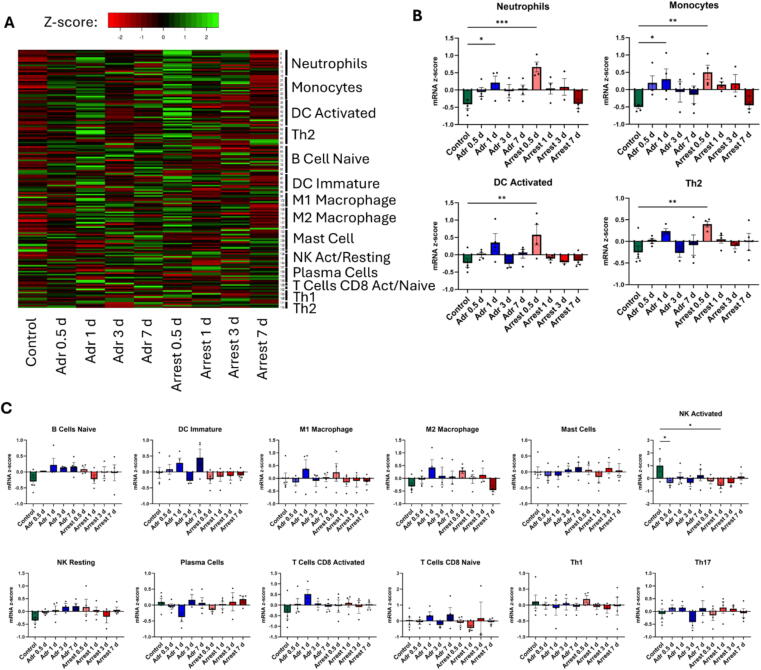
**Immunoprofiling of Adrenaline and Arrest hearts based on transcriptome data.** A. Heat maps demonstrating up- (green) or downregulation (red) of genes associated with 16 immune cell populations types based on z-scoring of gene expression for Adr (left) and Arrest (right) mice compared to Naïve controls. B. Comparison of average z-score change for neutrophils, monocytes, activated dendritic cells (DC), and T-helper 2 cells (Th2) comparing Adr and Arrest end-points to Naïve controls. C. Comparison of remaining immune cell populations between end-points and Naïve controls. *n* = 3–5/group. *=*p* < 0.05, **=*p* < 0.01, ***=*p* < 0.001 by ANOVA with Dunnett’s multiple comparison test. (For interpretation of the references to colour in this figure legend, the reader is referred to the web version of this article.)

In order to more deeply characterize the inflammatory changes implicated by RNA sequencing, immunoprofiling was completed on transcriptome data to assess specific immune cell groups contributing to bulk RNA changes. Sixteen immune cell populations were assessed by comparing gene profiles linked to specific populations and generating the z-scores of mRNA expression changes between treatment groups (Fig. 3A). Heat maps of these z-scores indicate a marked upregulation of immune cell-related genes, most prominently at 1 day in Adr mice compared to Naïve, and at 0.5 days in Arrest mice compared to Naïve. Neutrophil-associated genes were elevated in 0.5-day arrest mice [z-score 0.67 ± 0.14, *p* < 0.001] compared to Naïve mice [−0.41 ± 0.13] and in 0.5-day Adr mice [0.21 ± 0.19, *p* < 0.05] compared to Naïve. Monocyte-associated genes were similarly elevated in 0.5-day arrest mice [z-score 0.50 ± 0.21, *p* < 0.01] compared to Naïve mice [-0.50 ± 0.05], and in 0.5-day Adr mice [0.19 ± 0.2, *p* < 0.05] compared to Naïve. Additionally, dendritic cells (DCs) and T Helper 2 (Th2) cells were elevated in Arrest mice at 0.5 days compared to Naïve animals [DC z-score 0.58 ± 0.29 in Arrest compared to −0.24 ± 0.12 in Naïve, *p* < 0.05; Th2 z-score 0.40 ± 0.06 in Arrest compared to −0.25 ± 0.14 in Naïve, *p* < 0.05) No other immune cell groups showed significant elevations at any time point.

**Fig. 4:**
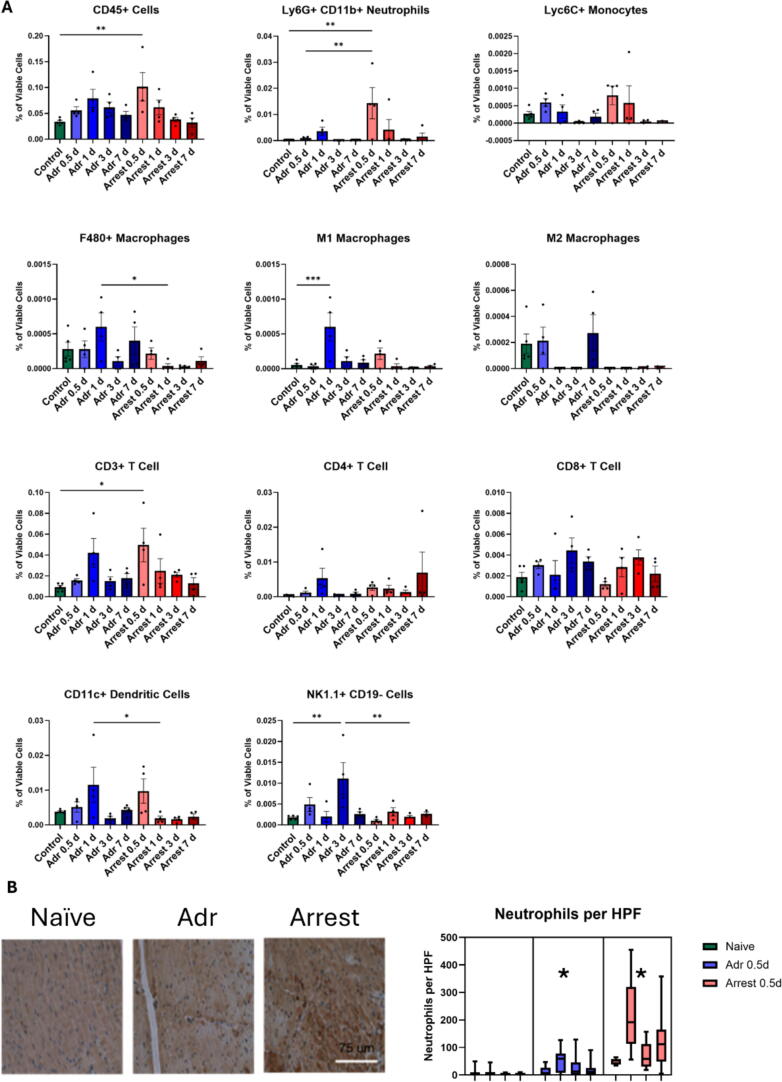
**Quantification of immune cell populations in Adr and Arrest hearts by flow cytometry.** A. Quantities represent the percent of viable cells that stained positive for markers of specific immune population. Comparisons are made between all end-points to Naïve controls as well as between same day Adr and Arrest end-points *n* = 3–5/group. *=*p* < 0.05, **=*p* < 0.01, ***=*p* < 0.001 by ANOVA with Dunnett’s multiple comparison test. B. Representative histologic sections with DAB staining of myeloperoxidase as a marker of neutrophil infiltration in Naïve controls as well as Adr and Arrest hearts from 0.5 d. *n* = 4/group. *=*p* < 0.05 compared to Naïve controls by nested 1 way ANOVA with Tukey’s multiple comparison test.

**Fig. 5:**
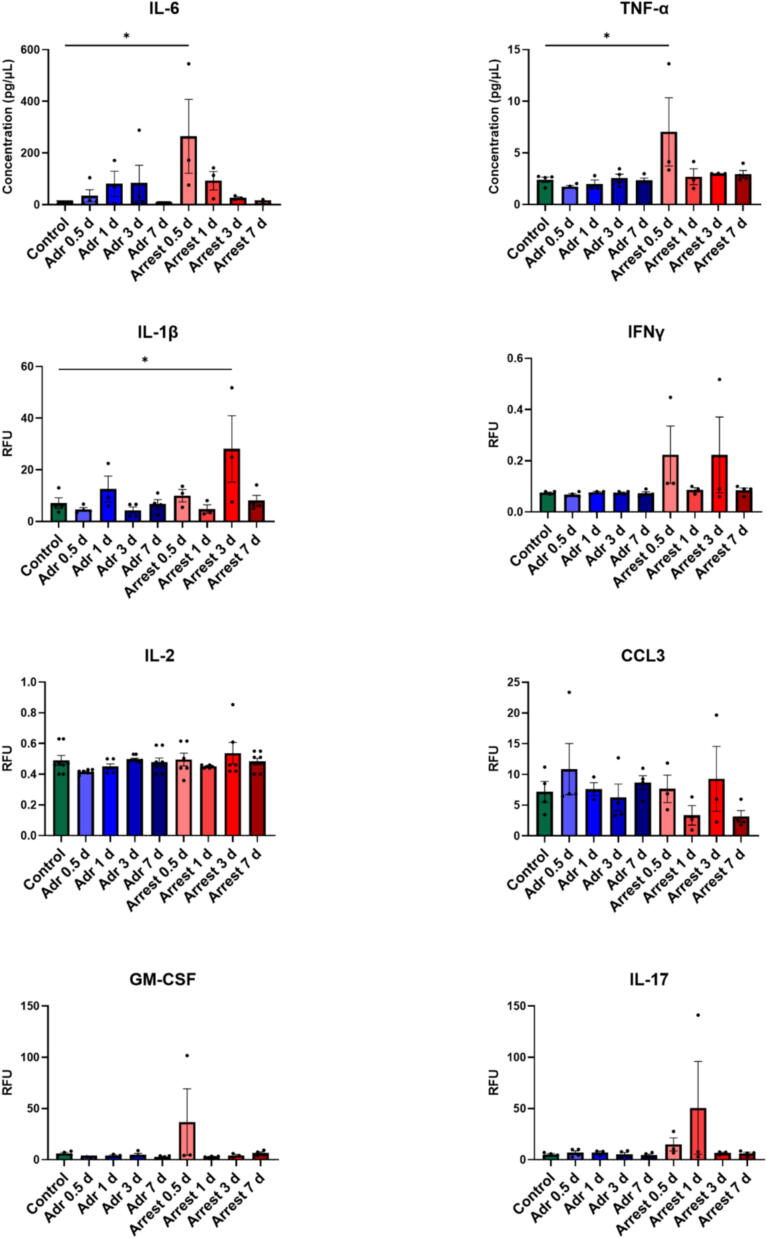
**Peripheral blood cytokine levels.** Blood from each end-point was evaluated for cytokine levels by multiplex assay kit for levels of interleukin 6 (IL-6), tumor necrosis factor α (TNF- α), interleukin 1β (IL1 β), interferon γ (IFN γ), interleukin 2 (IL2), chemokine ligand 3 (CCL3), granulocyte–macrophage colony-stimulating factor (GM-CSF), and interleukin 17 (IL-17). *n* = 3–5/group. *=*p* < 0.05 by ANOVA with Dunnett’s multiple comparison test.

To further define the cellular immune landscape of the heart following CA, we performed flow cytometry on dissociated myocardial tissue at serial time points post-intervention. Total CD45^+^ leukocytes were significantly elevated in the Arrest group at 0.5 days post-resuscitation [0.10 ± 0.03 %, *p* < 0.001] compared to Naïve [0.03 ± 0.00;Fig. 4A]. This elevation was primarily driven by an acute influx of neutrophils (CD11b^+^ Ly6G^+^; 0.014 ± 0.006 % in Arrest compared to 0.000 ± 0.000 in Naïve, *p* < 0.01). Neutrophil numbers peaked at 0.5 days and declined sharply in the Arrest group. Adr mice exhibited an increased concentration of macrophages at 1 day [0.0006 ± 0.0002 %, *p* < 0.01] compared to Arrest mice [0.000 ± 0.000] at the same time point, with elevated M1 macrophages [0.0006 ± 0.0002 % in Adr vs 0.000 ± 0.000 in Arrest, *p* < 0.01], specifically. 1-day Adr mice also showed elevated DCs compared to Arrest mice at the same time point [0.012 ± 0.005 % in Adr vs 0.002 ± 0.001 in Arrest, *p* < 0.01 ], and finally, natural killer (NK) cells were significantly elevated in 3-day Adr mice [0.011 ± 0.004, *p* < 0.01] when compared to both Naïve [0.002 ± 0.000] and 3-day Arrest mice [0.002 ± 0.000].

Histological Confirmation of Increased Neutrophil Infiltration in the Cardiac Tissues of Arrest Mice at 0.5 Days Post-Procedure.

Next, to validate neutrophil infiltration within the affected cardiac tissues in our CA model, histological staining was performed at 0.5 days post-procedure. This analysis confirms the observed increase in neutrophil presence, providing direct evidence of acute inflammatory cell recruitment following cardiac arrest. Our analysis revealed a significantly higher neutrophil count per high-powered field in the cardiac tissue of Arrest [117.6 ± 23.9 cells/HPF, *p* < 0.05] compared to Naïve mice [5.7 ± 2.9] and Adr mice [27.8 ± 10.1, *p* < 0.05] compared to Naïve mice (Fig. 4B). This increased neutrophil presence aligns with the Arrest immunoprofiling data, further validating the acute inflammatory response observed shortly after CA.

### Flow cytometry analysis revealed modulation of natural killer cells but not of other immune cells in the peripheral blood among the experimental groups

Flow cytometry analysis was performed on peripheral blood mononuclear cells (PBMCs) across groups to assess systemic immune cell alterations after CA. Notably, there was a significant decrease in natural killer (NK) cells in the Arrest mice at 1-day post-procedure [0.018 ± 0.004 %, *p* < 0.05] compared to Naïve mice [0.052 ± 0.006], indicating a potential suppression or redistribution of these cells in response to CA (Supplemental Fig. 1). Other immune cell populations remained consistent across the experimental groups, suggesting that the observed inflammatory response is primarily localized to specific immune cells without widespread alterations.

### Elevation of pro-inflammatory cytokine signaling in the acute phase in response to cardiac arrest

To investigate the systemic inflammatory response following CA, we conducted comprehensive cytokine profiling on plasma samples collected at various time points from all groups (Fig. 5). The analysis revealed a significant increase in pro-inflammatory cytokines IL-6 and TNF-α in the Arrest mice at 0.5 days post-procedure compared to Naïve controls [IL-6: 262.2 ± 143.1 pg/µl in Arrest vs 7.3 ± 1.5 in Naïve, *p* < 0.05; TNF-α 7.04 ± 3.3 pg/µl in Arrest vs 2.4 pg/µl0.3 in Naive, *p* < 0.05]indicating an acute inflammatory response shortly after CA. Additionally, the Arrest group at 3 days post-procedure exhibited elevated levels of IL-1β [28.1 ± 12.9 in Arrest vs 7.1 ± 2.1 in Naive, *p* < 0.05], suggesting a sustained but evolving inflammatory response. However, no other cytokines showed significant changes between the groups at any of the time points examined, highlighting the specificity of these pro-inflammatory cytokines in the early stages of post-arrest inflammation. This cytokine profile underscores the role of IL-6, TNF-α, and IL-1β as key mediators of the peripheral inflammatory response following CA.

## Discussion

CA is known to initiate a broad cascade of pathophysiological events, including myocardial dysfunction, systemic inflammation, and metabolic derangements, that contribute to the high morbidity and mortality seen in survivors.^16^ While early post-resuscitation myocardial dysfunction is well described in both preclinical and clinical settings,^26^, ^27^ the underlying inflammatory processes within the myocardium remain incompletely understood. In this study, we provide a detailed characterization of the myocardial inflammatory response following cardiac arrest (CA) and resuscitation in mice. We demonstrate that CA triggers a rapid and robust inflammatory response in the heart, characterized by early neutrophil infiltration and marked systemic upregulation of IL-6 and TNF-α. These changes coincided with a reduction in LV EF, indicating a close temporal relationship between immune activation and myocardial dysfunction. The inflammatory response was transient; both immune cell infiltration and transcriptomic perturbations returning to baseline by day 7, paralleling recovery of systolic function. Finally, we show that adrenaline exposure alone produced only a delayed and blunted inflammatory response, confirming that ischemia–reperfusion rather than catecholaminergic stimulation is the primary driver of myocardial immune activation.

Immunoprofiling and flow cytometry confirmed a coordinated immune cell response in the post-arrest heart. The early influx of neutrophils into myocardial tissue and upregulation of IL-6 and TNF-α align with classical paradigms of sterile inflammation seen in ischemic injury.^28^ This acute inflammatory surge, while potentially beneficial for debris clearance and repair signaling, may also contribute to secondary myocardial injury through oxidative stress, extracellular trap formation, and microvascular dysfunction.^29^ Neutrophils are known mediators of reperfusion injury through oxidative damage and protease release, and their presence has been associated with ventricular dysfunction in both animal and human models; unfortunately, neutrophil depletion has not improved outcomes in prior CA models.^30^, ^31^ Adr mice exhibited a delayed and attenuated neutrophilic response, consistent with reported detrimental effects of adrenaline treatment.^14^, ^32^, ^33^

We also observed elevated dendritic cells and Th2-associated transcripts early after CA, indicating a mild evolving adaptive immune response. These changes were paralleled by cytokine shifts in the systemic circulation, with early increases in IL-6 and TNF-α at 0.5 days, followed by sustained IL-1β at day 3. Clinical studies of out-of-hospital CA patients have found that elevated IL-6 levels correlate with 30 day mortality.^34^ IL-1β and TNF-α have also been identified as altered biomarkers in CA survivors, but have not demonstrated reliable association with longer term mortality.^35^, ^36^ Recent clinical trials have targeted similar inflammatory pathways. For example, IL-6 blockade (tocilizumab) has shown potential to blunt systemic inflammation and improve hemodynamics in post-CA patients,^37^ while corticosteroids may reduce post-resuscitation shock and inflammatory burden.^38^ Similarly, TNF-α blockage improves outcomes in a pig model.^39^ Our findings provide mechanistic support for these interventions by confirming robust and early cytokine activity in the post-arrest mouse.

The resolution of transcriptional and immunologic changes by day 7 in both groups suggests that the inflammatory response is self-limited in the absence of structural heart disease. This temporal pattern highlights a potential therapeutic window during which interventions to modulate inflammation, particularly targeting neutrophil infiltration or cytokine signaling, may be most effective. Importantly, we found little evidence of macrophage expansion or widespread systemic immune activation, suggesting that the immune response is primarily localized and short-lived.

From a translational standpoint, this study offers several key insights. First, the reproducible timing of inflammation and cardiac recovery provides a potential window for immunomodulatory intervention. Second, the delineation of ischemia-specific versus adrenaline-mediated responses highlights the importance of context when interpreting immune signatures in cardiac injury. Finally, the use of an integrated multi-modal platform, combining echocardiography, RNA sequencing, immunophenotyping, and histology, offers a blueprint for comprehensive cardiac immune profiling in both preclinical and clinical settings.

### Limitations and future directions

Several limitations warrant discussion. While this murine model recapitulates essential features of human CA, including transient systolic dysfunction and sterile inflammation, species-specific differences in immune kinetics must be considered when extrapolating to human physiology. The bulk tissue approach does not resolve cell-type specific transcriptomic changes, and future studies may utilize single-cell transcriptional analysis to more deeply categorize immune subtypes or spatial transcriptomic analyses to better determine regional immune changes in the heart. There are specific limitations to our mouse model. First, all treatment groups were exposed to isoflurane anesthesia, which can alter systemic and local inflammatory changes in the mouse,^40^, ^41^ potentially confounding our findings. Second, we do not include invasive hemodynamic monitoring, rather utilizing echocardiography to non-invasively monitor cardiac function. Finally, while intraventricular injection of KCl and adrenaline ensures reproducible induction and resuscitation, it introduces the possibility of local myocardial injury unrelated to systemic ischemia or catecholaminergic stimulation. To reduce this potential confounding, we excluded apical tissue adjacent to the injection site from analysis; however, we acknowledge that this approach may not fully eliminate injection-related bias. While our study focused on the acute immune phase, longer-term consequences of cardiac inflammation on fibrosis, remodeling, and arrhythmogenesis remain to be explored.

## Conclusions

Cardiac arrest triggers a rapid and transient myocardial inflammatory response marked by neutrophil infiltration, cytokine release, and transcriptional reprogramming. These changes coincide with myocardial stunning and resolve by one-week post-resuscitation. Our findings support the hypothesis that early innate immune activation contributes to post-arrest myocardial dysfunction and suggest that therapeutic strategies targeting inflammation, particularly within the first 24 h, may improve cardiac recovery and survival. These findings uncover a narrow but actionable therapeutic window and support the development of precision immunotherapies aimed at mitigating myocardial injury and improving post-arrest outcomes.

## CRediT authorship contribution statement

**Soumya Panigrahi:** Writing – review & editing, Writing – original draft, Visualization, Investigation, Formal analysis. **Siyi Jiang:** Writing – review & editing, Investigation. **Angela Enriquez:** Writing – review & editing, Investigation. **Sanjana Tummala:** Writing – review & editing, Investigation, Formal analysis. **Donald Rempinski:** Writing – review & editing, Investigation. **Kenneth E. Remy:** Writing – review & editing, Writing – original draft. **Cody A. Rutledge:** Writing – review & editing, Writing – original draft, Visualization, Supervision, Methodology, Investigation, Funding acquisition, Formal analysis, Conceptualization.

## Declaration of competing interest

None of the authors have financial disclosures or conflicts of interest.
