# Supplementary material for: Temporal profiling of myocardial inflammation and recovery in a murine model of cardiac arrest and adrenaline exposure
Source: Resusc Plus. 2025 Oct 17;26:101137. doi: 10.1016/j.resplu.2025.101137 (PMC12744645; doi:10.1016/j.resplu.2025.101137)
Supplement: Supplementary Data 1 [file mmc1.pdf]

Supplement to "Temporal profiling of myocardial inflammation and recovery in a murine model of cardiac arrest and adrenaline exposure"

Soumya Panigrahi MBBS PhD[1], Alice Jiang BS[2], Angela Enriquez BA[2], Sanjana Tummala[1], Donald Rempinski BA[1], Kenneth E. Remy MD, MHSc, MSCI, FCCM[1], Cody A. Rutledge MD PhD[1-4]

1. Blood, Heart, Lung, and Immunology Research Center, Department of Medicine, Case Western Reserve University School of Medicine, Cleveland, Ohio, USA
2. Division of Cardiology, Vascular Medicine Institute, Department of Medicine, University of Pittsburgh, Pittsburgh, PA, USA
3. Department of Medicine, Louis Stokes Cleveland VA Medical Center, Case Western Reserve University School of Medicine, Cleveland, Ohio, USA
4. Department of Medicine, Pittsburgh VA Medical Center, Pittsburgh, PA, USA

Corresponding Author:
Dr. Cody Rutledge
Wolstein Research Building 4th Floor, Room 4501A
2103 Cornell Rd
Cleveland, OH, 44106
Email: car24@case.edu
216-791-3800

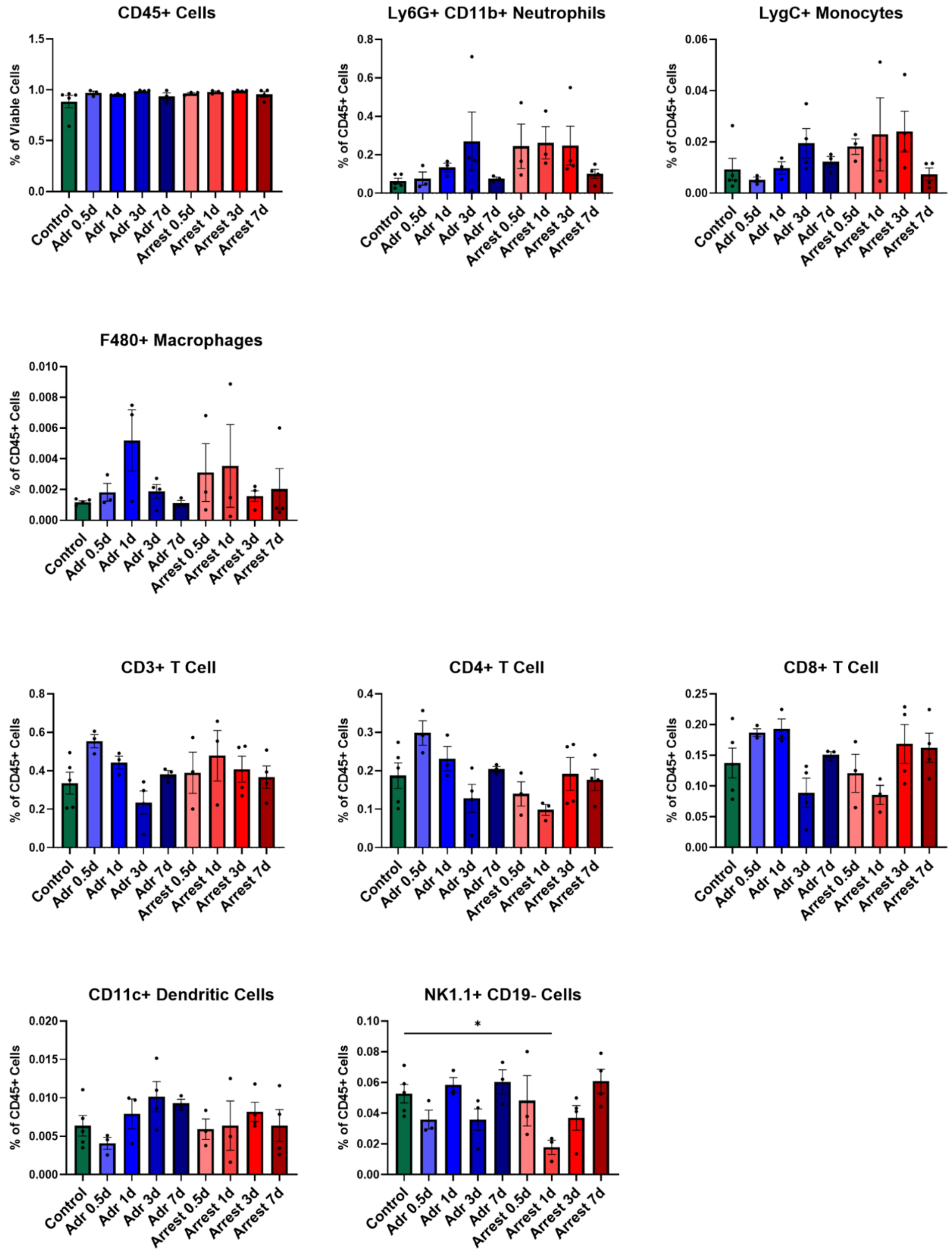

Supplemental Figure 1. Quantification of immune cell populations in Adrenaline (Adr) and Arrest peripheral blood by flow cytometry. Quantities represent the percent of viable cells that stained positive for markers of specific immune population. Comparisons are made between all end-points to Naïve controls as well as between same day Adr and Arrest end-points. A full gating strategy is available in Supplementary Figures 3-4. n=3-5/group. *=p <0.05 by ANOVA with Dunnett's multiple comparison test.

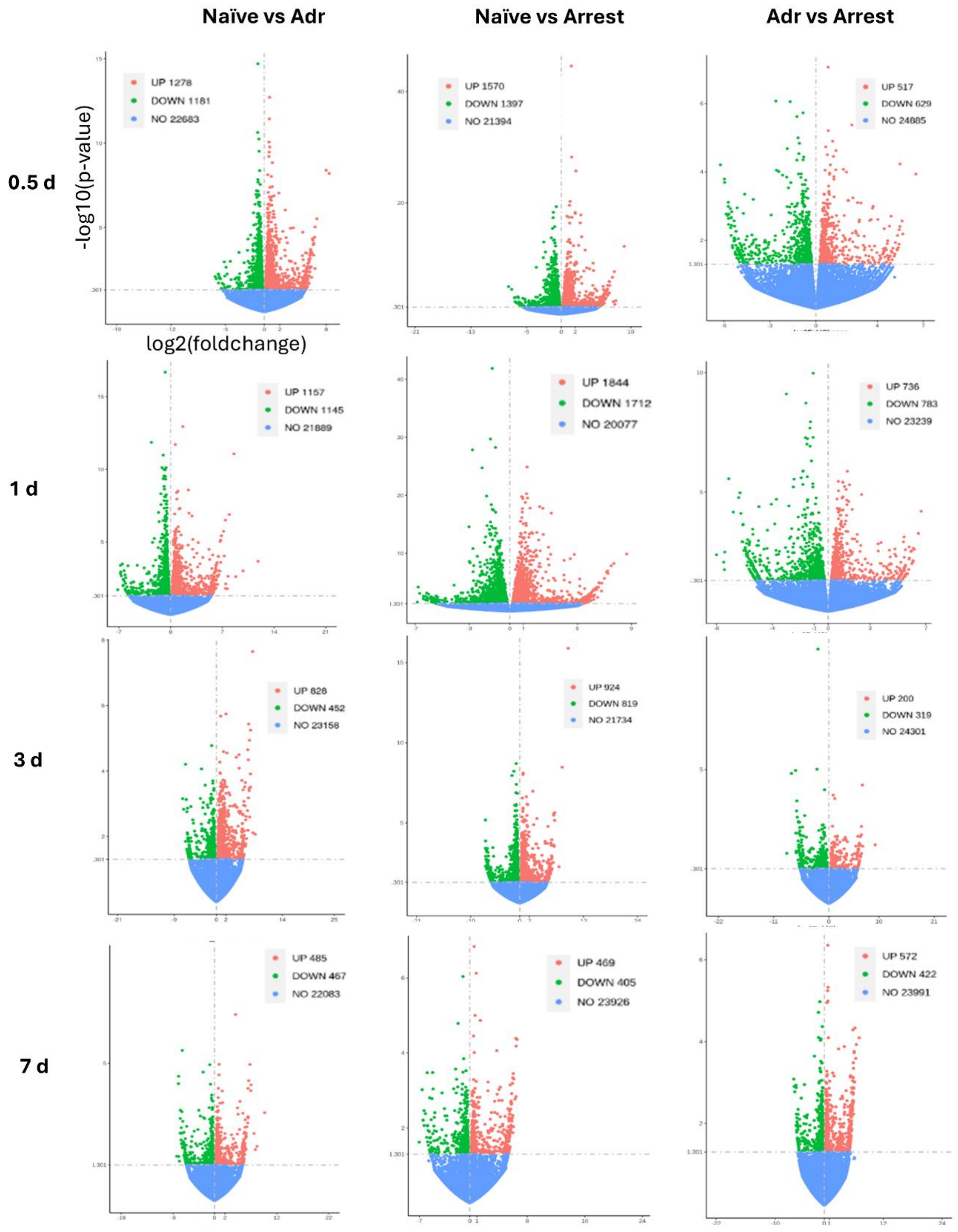

Supplemental Figure 2. Volcano plots of differentially expressed genes between groups. All Adr and Arrest end-points are compared to a single group of Naïve controls. Adr vs Arrest groups are compared at the same endpoint (i.e. 0.5 d sham vs 0.5 d arrest).

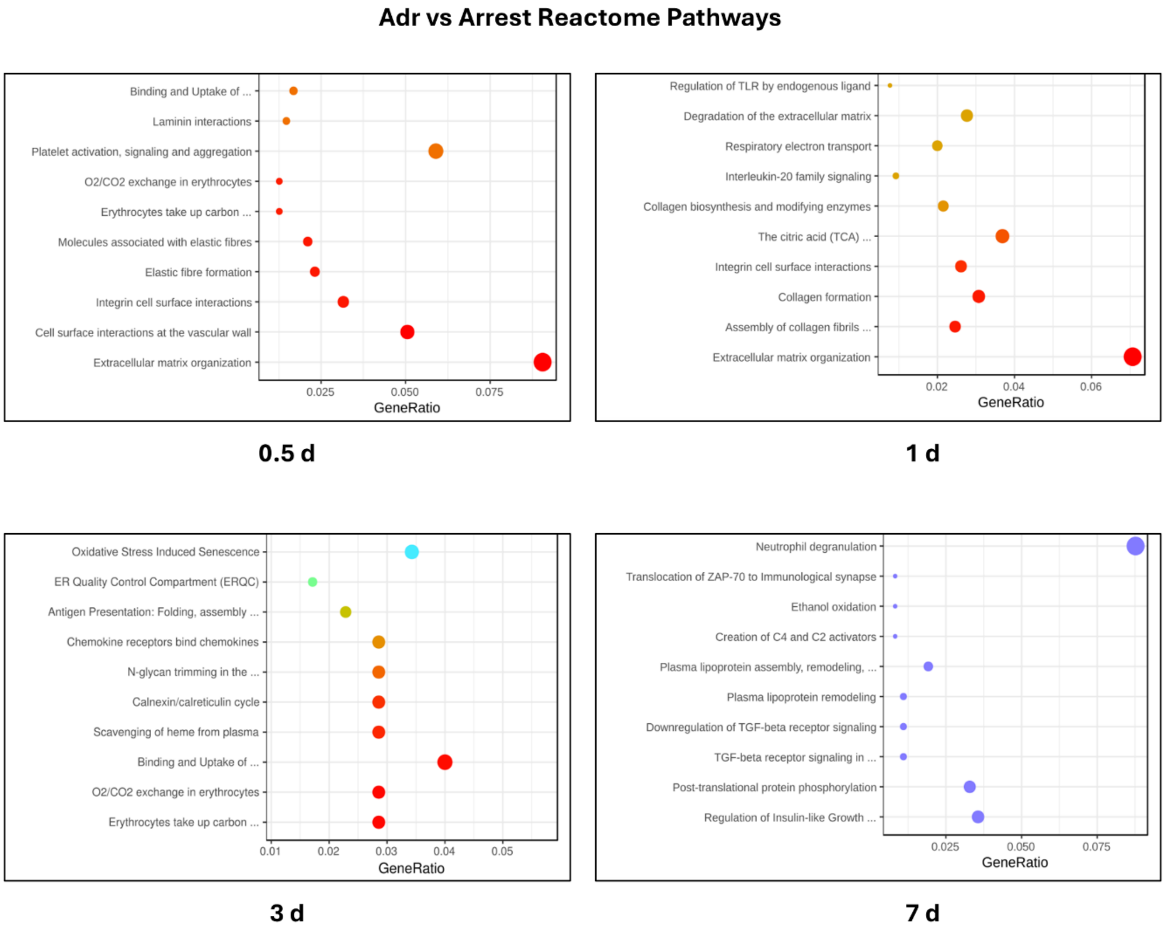

Supplemental Figure 3. Pathway Analysis of Adr and Arrest myocardial transcriptomes at each end-point. The top 10 Reactome pathway analyses comparing Adr and Arrest hearts at study end-points.

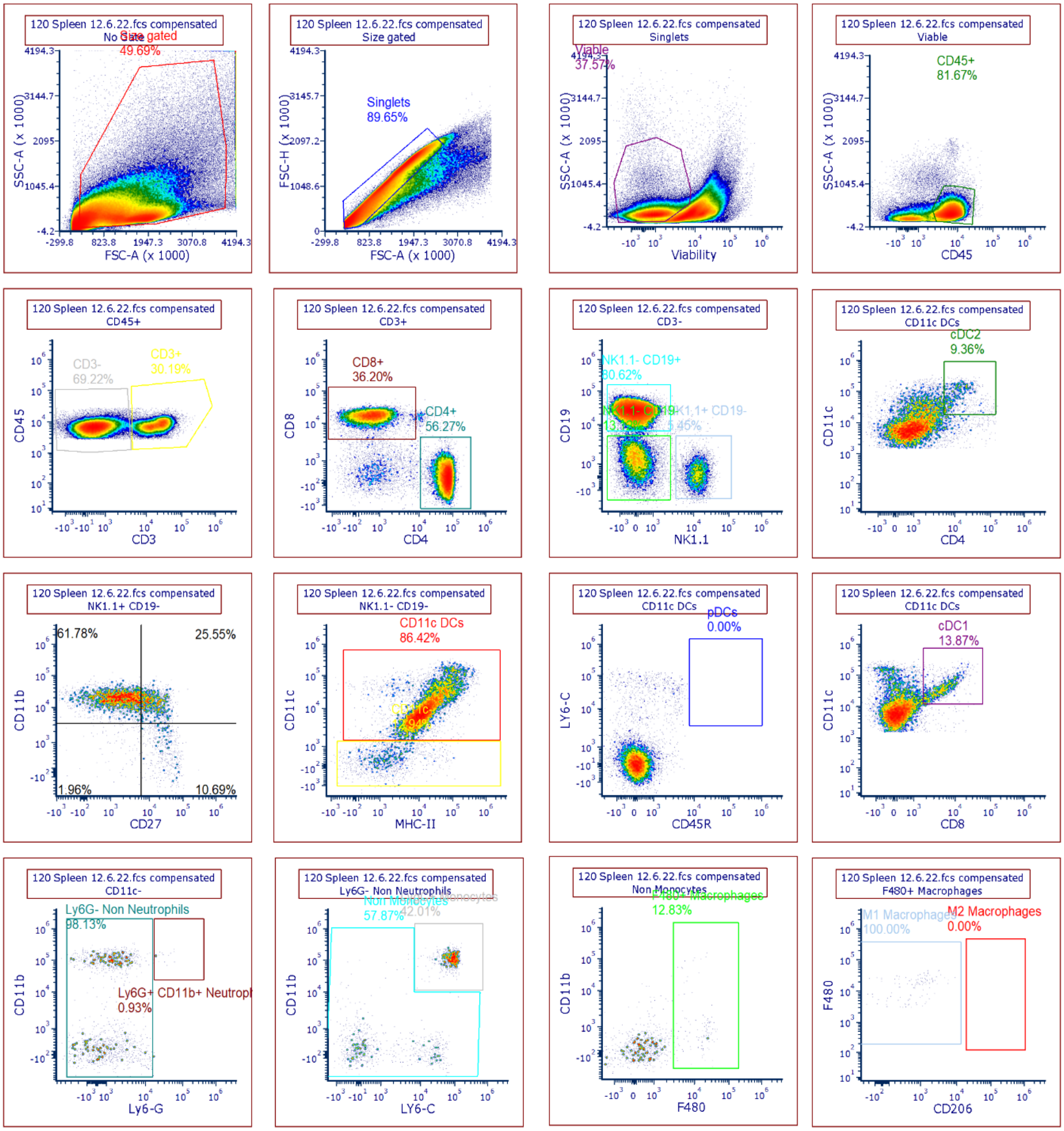

Supplemental Figure 4. Gating strategy for infiltrating immune cells taken from a representative spleen.

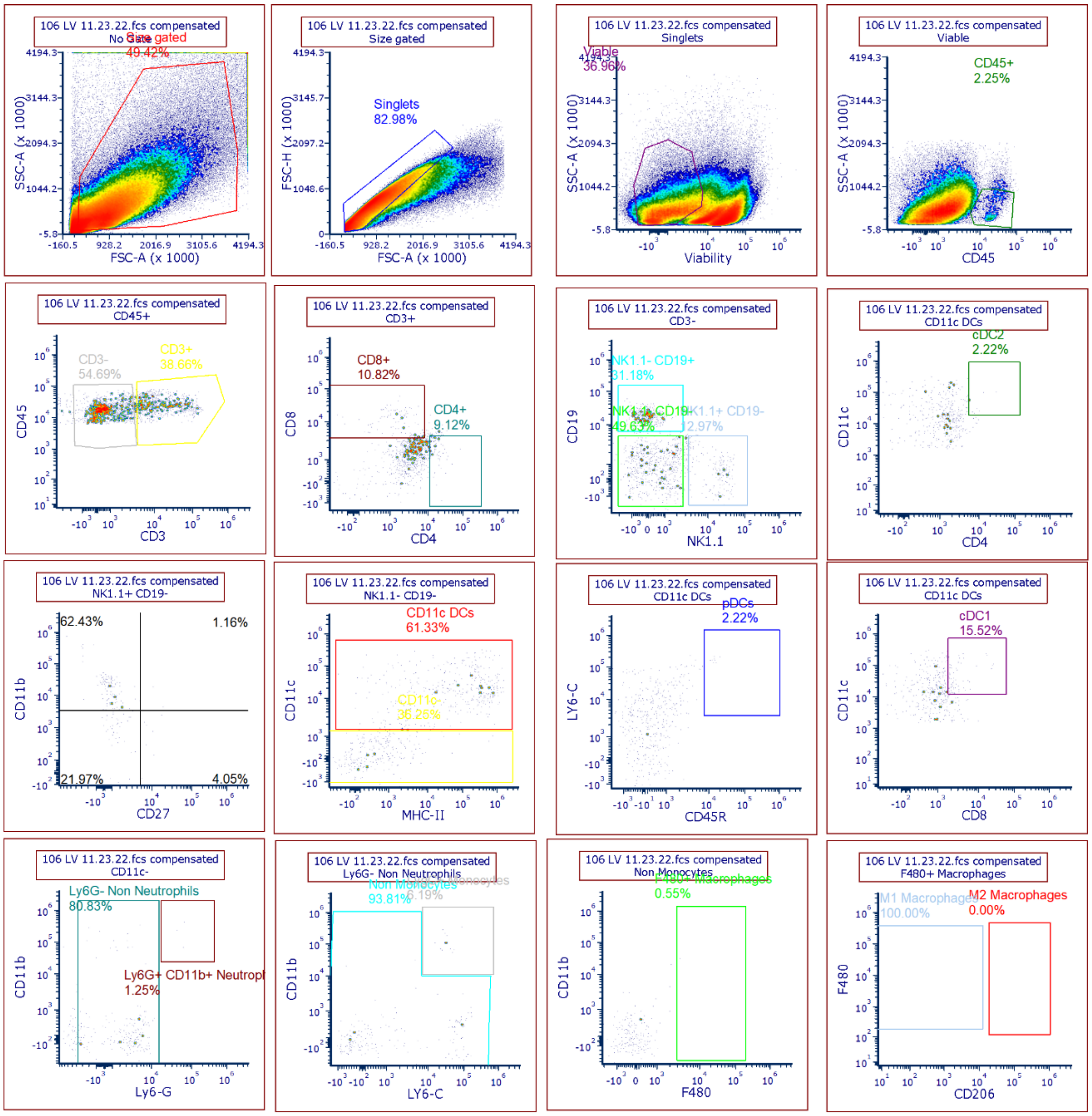

Supplemental Figure 5. Gating strategy for infiltrating immune cells taken from a representative left ventricle.

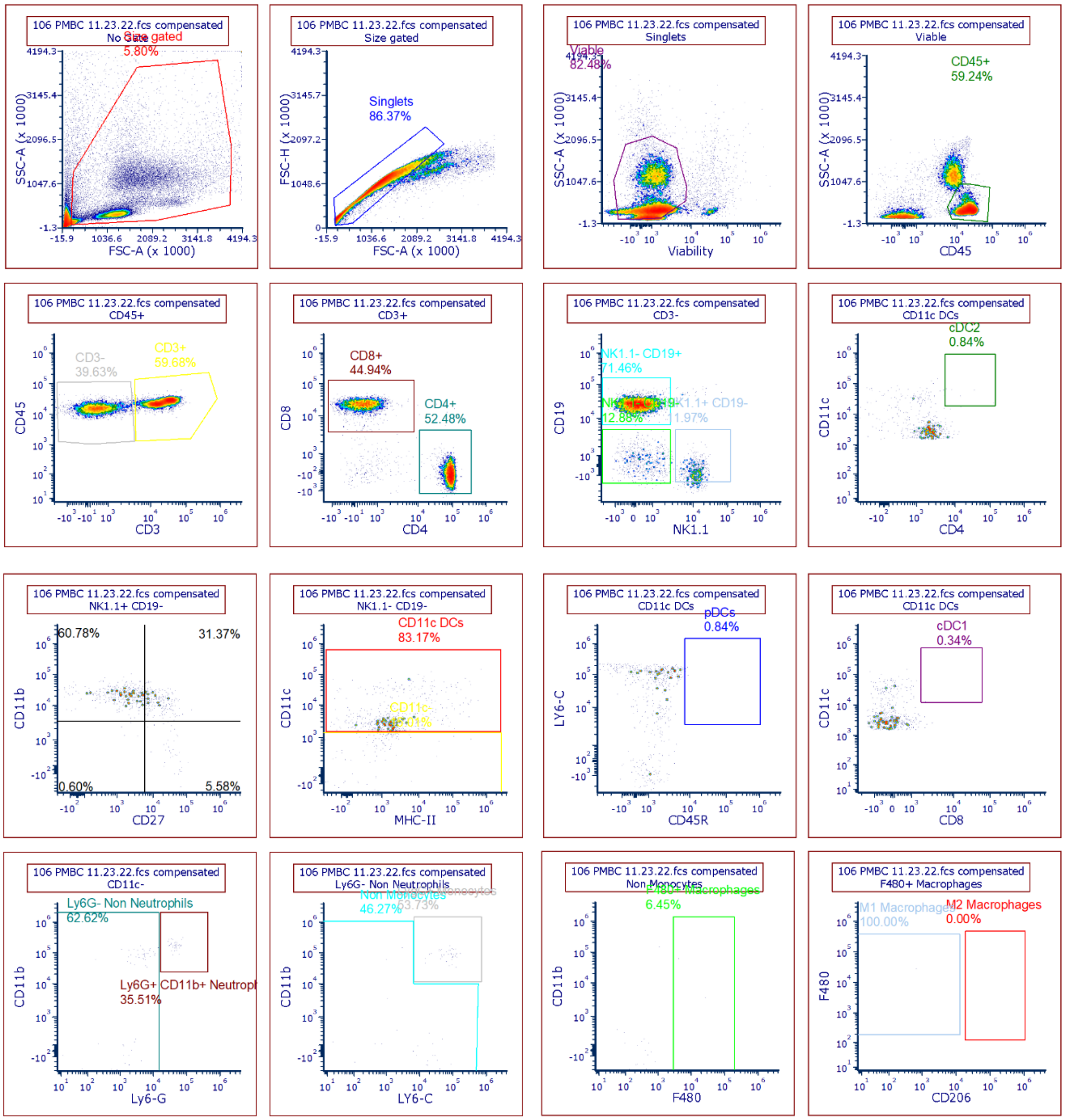

Supplemental Figure 6. Gating strategy for immune cells taken from peripheral mononuclear blood cells.
